# Screening behaviors for diabetic foot risk and their influencing factors among general practitioners: a cross-sectional study in Changsha, China

**DOI:** 10.1186/s12875-023-02027-3

**Published:** 2023-03-13

**Authors:** Nan Zhao, Jingcan Xu, Qiuhong Zhou, Juanyi Hu, Wenjing Luo, Xinyi Li, Ying Ye, Huiwu Han, Weiwei Dai, Qirong Chen

**Affiliations:** 1 Teaching and Research Section of Clinical Nursing, Xiangya Hospital of Central South University, 410008 Changsha, China; 2 Department of Nursing, The Second Affiliated Hospital of Xi’an Jiaotong University, 710004 Xi’an, Shaanxi Province China; 3 National Clinical Research Center for Geriatric Disorders, Xiangya Hospital, Central South University, 410008 Changsha, Hunan China; 4 Xiangya School of Nursing, Central South University, 410013 Changsha, China; 5 Department of Stoma Wound Care Center, Xiangya Hospital, Central South University, 410008 Changsha, China

**Keywords:** General practitioner, Diabetic foot, Risk screening, Behavior

## Abstract

**Background:**

Diabetic foot is a serious complication of diabetes with a high disability and mortality rate, which can be prevented by early screening. General practitioners play an essential role in diabetic foot risk screening, yet the screening behaviors of general practitioners have rarely been studied in primary care settings. This study aimed to investigate foot risk screening behaviors and analyze their influencing factors among general practitioners.

**Methods:**

A cross-sectional study was conducted among 844 general practitioners from 78 community health centers in Changsha, China. A self-designed and validated questionnaire was used to assess the general practitioner’s cognition, attitude, and behaviors on performing diabetic foot risk screening. Multivariate linear regression was conducted to investigate the influencing factors of risk screening behaviors.

**Results:**

The average score of diabetic foot risk screening behaviors among the general practitioners was 61.53 ± 14.69, and 271 (32.1%) always or frequently performed foot risk screening for diabetic patients. Higher training frequency (*β* = 3.197, *p* < 0.001), higher screening cognition (*β* = 2.947, *p* < 0.001), and more positive screening attitude (*β* = 4.564, *p* < 0.001) were associated with more diabetic foot risk screening behaviors, while limited time and energy (*β*=-5.184, *p* < 0.001) and lack of screening tools (*β*=-6.226, *p* < 0.001) were associated with fewer diabetic foot screening behaviors.

**Conclusion:**

The score of risk screening behaviors for the diabetic foot of general practitioners in Changsha was at a medium level. General practitioners’ diabetic foot risk screening behaviors may be improved through strengthening training on relevant guidelines and evidence-based screening techniques, improving cognition and attitude towards foot risk screening among general practitioners, provision of more general practitioners or nurse practitioners, and user-friendly screening tools.

## Background

Diabetic foot consists of infection, ulceration, and destruction of the foot tissues that are mainly caused by peripheral neuropathy and peripheral arterial disease [1, 2]. Diabetic foot ulcer (DFU) is one of the most common and devastating chronic complications of diabetes, with a global prevalence of 6.3% among diabetic patients. DFU is the leading cause of hospitalization, amputation, reduced mobility, loss of social participation, and lower quality of life in people with diabetes [3]. It has been reported that every 20 s in the world a lower limb was amputated due to a diabetic foot, and the mortality rate after amputation is as high as 50% [5–7]. Additionally, diabetic foot causes tremendous physical and psychological suffering to the patient and place a huge burden on the individual, family, and society due to increased healthcare costs. The disease burden of diabetic foot is ranked in the top 10 of all medical conditions [4].

Prevention of diabetic foot is more important than treatment. Regular screening and timely identification of risk factors for diabetic foot is the most cost-effective way to prevent diabetic foot. Studies have demonstrated that early risk identification and prevention can prevent half of diabetic patients from developing foot ulcers or amputations [6, 7]. It is thus crucial to carry out diabetic foot screening to facilitate early detection, prevention, diagnosis, and treatment of diabetic foot [8–12]. The American Diabetes Association (ADA) guidelines recommend [13] that all patients with diabetes should be screened for risk factors of ulcers and amputation, the feet should be checked at each visit, and a comprehensive foot assessment should be performed at least once a year. The International Working Group on the Diabetic Foot’s (IWGDF) “Practical Guidelines on the Prevention and Management of Diabetic Foot Disease (2019)” also gives clear recommendations on the content and frequency of diabetic foot risk screening and provides a risk grading system for diabetic foot [14].

However, the overall status of screening is not optimistic, the foot risk screening rate of diabetic patients in different countries ranges from 15.7 to 64.8% [15–17]. A multi-center cross-sectional study in Spain found that 56.4% of patients with diabetes underwent foot screening, of which 39.5% underwent 10 g monofilament (10 g-MF) exploration, 45.8% underwent palpation of the dorsal foot artery, and 10.1% received ankle-brachial index test [15]. A Canadian survey of 13,388 people with diabetes showed that 7,277 (53%) had at least one foot exam by a healthcare provider in the past year [16]. According to the Scottish Diabetes Survey from 2019, 56.7% of people with type 1 diabetes and 64.8% with type 2 diabetes had received foot risk screening within 15 months [17]. Compared with these countries, Australia has a lower rate of foot screening for diabetics, a study for Primary Care found that only 45% of people with diabetes said they would take off their diabetic shoes and socks for a foot risk screening when visiting a doctor [18]. Studies in different countries have shown that primary medical staff play a very important role in the foot risk screening of diabetic patients [15–17]. China has the largest population with diabetes in the world. It is reported that 57.1% of Chinese diabetic patients were at high risk of diabetic foot, yet only 15.7% of them have performed regular foot risk screening, a huge gap exists between the high-risk and low risk screening rates of diabetic foot among diabetic patients [19]. In recent years, the number of general practitioners in primary care settings in China has gradually increased due to policy encouragement [20], as the main force of primary medical institutions, general practitioners act as the goalkeeper for the implementation of primary prevention of diabetic foot and thus are required to master diabetic foot risk screening [15, 17, 20].

Although general practitioners play a vital role in diabetic foot risk screening, we found only a few surveys on community health care workers’ perceptions of diabetic foot screening [21], yet little is known about whether and how general practitioners implemented diabetic foot risk screening among diabetic patients. To the best of our knowledge, only one study has investigated the rate of foot screening practiced by general practitioners. However, this study just asked general practitioners whether to perform diabetic foot ulcer screening and ignored the contents and methods of specific screening. There is also a paucity of studies on the influencing factors of risk screening behaviors for diabetic foot among general practitioners. Meanwhile, we also did not find studies related to the specific risk screening behavior for diabetic foot and its influencing factors among other health practitioners. Understanding the status of foot risk screening behavior and its influencing factors is of great importance for constructing foot screening interventions and improving the awareness of foot screening for diabetic patients among primary medical staff [15–17]. At the same time, it provides a theoretical basis for improving foot risk screening behavior. Therefore, this study aimed to understand the current level and specific screening behavior of diabetic foot risk screening for diabetic patients by general practitioners in Changsha, China, and to analyze its influencing factors. The findings of our study will provide useful and important guidance for improving diabetic foot risk screening for diabetic patients in primary care, so as to prevent the occurrence of diabetic foot and improve the outcomes of diabetic patients.

## Methods

### Ethics approval

The study received ethical approval from the Institutional Review Board of the Xiangya Hospital Medicine Ethics Committee, Central South University [202,103,024] and all participants provided consent to participate online.

### Study design, participants, and procedure

This study conducted a cross-sectional study in Changsha, Hunan Province of China from April 12 to 20, 2021. Our target population was all general practitioners working in all community health centers in Changsha City, Hunan Province, China. The inclusion criteria included: (1) Engaged in diabetes management for more than 1 year; (2) Qualified as a general practitioner. The Exclusion criteria included: (1) Failed to work normally due to illness, pregnancy, or other reasons; (2) Refused to be investigated.

The study was approved by the Institutional Review Board of the Xiangya Hospital Medicine Ethics Committee, Central South University (No. 202,103,024), and was in accordance with the Declaration of Helsinki. The survey was conducted through an online survey platform named the “questionnaire star” with a QR code link, with the support of the administrative department in charge of managing the community health service center in Changsha, China, we obtained approval and help from each of the 78 community health centers, then we sent the QR code link to an online questionnaire to eligible general practitioners via Wechat (Most widely used social app in China). All participants were informed of the purpose, benefits, risks, and significance of the study, and gave electronic informed consent. Participation was voluntary, and responses were anonymous. In the meantime, this online questionnaire improves the quality of the questionnaire collection by setting up mandatory questions, limiting the target object, and submitting the same IP address only once.

Finally, we approached 964 general practitioners to participate in the online questionnaire and received 867 questionnaires. After excluding 23 invalid questionnaires with response time less than 180s (according to the data of the pilot test), we finally received 844 valid questionnaires, with a response rate of 87.55%.

### Questionnaire

#### General Information Questionnaire

A self-designed general information sheet was used to collect the general practitioner’s basic demographic information such as gender, age, years of working, and education. We also asked about the training frequency that the general practitioner received on diabetic foot risk screening in the last two years. In addition, we asked about the potential barriers the general practitioner may encounter that prevent them from performing diabetic foot risk screening on their diabetic patients, which included limited time and energy, lack of screening tools, expenses not covered by health insurance, and patients not cooperating.

#### Diabetic Foot Risk Screening Cognitive, attitudinal, behavioral questionnaire

Because there was no mature scale before. A self-designed Diabetic Foot Risk Screening Cognitive, Attitudinal and Behavioral Questionnaire was used to assess the general practitioner’s cognition, attitude, and behaviors on performing diabetic foot risk screening on diabetic patients in their routine work. The questionnaire was developed based on the International Working Group on the Diabetic Foot (IWGDF) [14] “Practical Guidelines on the prevention and management of diabetic foot disease (2019)”, and the “Consensus on Diabetic Foot Basic-level Screening and Prevention in China (2019) " [20]. The questionnaire was evaluated by experts familiar with the subject and purpose of the questionnaire. We recruited 5 experts, including 3 general practitioners and 2 experts in the field of diabetic foot, all of whom have worked for more than 10 years. Experts assessed whether the information was understandable and whether there was any improvement needed. After all the modifications were completed, the questionnaire will be sent back to experts to review whether the items were essential, useful, or unnecessary. For that purpose, experts assessed each item on a 4-point Likert scale ranging from 1 to 4 for clarity and relativity. Once this was done content validity indexes (CVI) were calculated for each question. The CVI of each item, both for relevance and clarity, was calculated by the ratio of the number of responses “3” or “4” in relation to the total number of responses to the item. The content-related validity of the item (CVI) reached 0.80. The scale-level content validity item (S-CVI) was 0.80. The questionnaire was also pilot-tested among a sample of 135 community general practitioners and demonstrated good internal consistency, with a Cronbach’α coefficient of 0.77.

The questionnaire consists of 15 items under three subscales: cognition, attitude, and behavior, it contains single-choice or multiple-choice, and the scores for each item are standardized, with 4 points for each item; 4 points for correct answers to multiple-choice questions, and 0 points for incorrect answers, the score of multiple-choice questions = (actual number of correct options/total number of correct options) × 4. The score of cognition of diabetic foot screening and behavior of diabetic screening transformed into a standard score for better comparison, using the following formula: standard score = (actual score/ highest possible score) × 100. The standard score was further classified into three categories: <60 as poor, 60–79 as medium, and > 80 as good [22].

*Cognition of diabetic foot risk screening* There are 9 items in the cognition subscale that assess the respondents’ knowledge and awareness of diabetic foot risk screening including screening objects and frequency, screening content and methods, and the knowledge of patient foot self-management. Among them, items 1–5 were “yes-no” questions, with each “yes” answer assigned 4 points, and each “no” answer assigned 0 point. The 6-9th item is a multiple-choice question, each chosen method was scored 4 points. The original total score of the cognition subscale ranges from 0 to 36.

*The attitude towards diabetic foot risk screening* The attitude towards diabetic foot risk screening was assessed by one question asking whether the respondents thought it necessary to carry out diabetic foot screening among diabetic patients in the communities, with optional answers being “strongly necessary”, ‘necessary” and “not necessary”.

*The behavior of diabetic foot risk screening* There are 5 items in the behavior subscale that assess the respondents’ diabetic foot risk screening behaviors in their routine practice. Including screening frequency, the content and methods of risk screening, information risk level, guidance for regular screening or referral, and foot self-management education. The item about respondents’ behavior of foot screening content is a multiple-choice question, and the rest items were rated on a 5-point Likert scale from 0 “occasionally/never” to 4 “always”. In addition, the commonly used methods of peripheral neuropathy and peripheral vascular lesions are not scored because there are no clear and correct answers. The original total score of the behavior subscale ranges from 0 to 20.

The questionnaire took approximately 4 min to complete, and detailed information on the questionnaire was listed in Supplement 1.

### Data analysis

All data analyses were conducted using SPSS 26.0. Continuous variables were described by means (standard deviations) for normal distributions and medians (quartiles) for non-normal distributions. Two independent sample t-tests and one-way analysis of variance were used for group comparisons, and significant results from univariate analysis were included in multiple linear regression analysis to explore factors influencing diabetic foot risk screening behavior. P values were 2-tailed and p < 0.05 was considered to be statistically significant.

## Results

### Diabetic foot screening behaviors

The average score of the diabetic foot risk screening behaviors was 61.53 ± 14.69, which was at a medium level overall. The specific implementation is shown in Table 1.

Table 2 further shows the tools and methods used for peripheral neuropathy and peripheral vascular disease. For peripheral neuropathy, the top 3 most frequently used methods were asking about symptoms (59.6%), pinprick (55.7%), and temperature sense (31.3%), while the top 3 least frequently used methods were Ipswich Touch Test (IPTT) (15.0%), Sensory threshold determination (8.3%), and Nerve conduction rate test (4.1%). For peripheral vascular disease, the most used method was palpating the dorsal/posterior tibial artery (50.9%), while the least used method was angiography (11.5%).

**Table 1:** Implementation of routine risk screening for diabetic foot

Screening items	Implementation rate
Screening Frequency (times)	
Always	64 (7.6%)
Frequently	207 (24.5%)
Sometimes	329 (39.0%)
Rarely	239 (28.3%)
Never	5 (0.6%)
**Screening content**	
Blood sugar	750 (88.9%)
History of diabetes comorbidities/complications	639 (75.7%)
General information	634 (75.1%)
Smoking history	622 (73.7%)
Foot skin	599 (71.0%)
Peripheral neuropathy	568 (67.3%)
Foot hygiene	585 (69.3%)
History of foot ulcer/amputation (toe)	571 (67.7%)
Habits of wearing shoes and socks	517(61.3%)
Peripheral vascular disease	512 (60.7%)
Foot care education	498 (59.0%)
Ankle joint activity	489 (57.9%)
Foot deformity	435 (51.5%)
**Inform risk level**	
Always	205 (24.3%)
Frequently	314 (37.2%)
Sometimes	195 (23.1%)
Rarely	112 (13.3%)
Never	18 (2.1%)
**Guidance for regular screening or referral**	
Always	239 (28.3%)
Frequently	293 (34.7%)
Sometimes	213 (25.2%)
Rarely	77 (9.1%)
Never	22 (2.6%)
**Foot self-management education**	
Always	152 (19.2%)
Frequently	226 (26.8%)
Sometimes	202 (23.9%)
Rarely	156 (18.5%)
Never	98 (11.6%)

**Table 2:** Methods used for screening peripheral neuropathy, peripheral vascular disease

Item	N (%)
Peripheral Neuropathy Screening Methods	
Ask about symptoms	503(59.6)
Pin prick	470(55.7)
Temperature sense	264(31.3)
Ankle reflex	259(30.7)
10 g-MF	203(24.1)
Tuning fork 128 Hz	148(17.5)
IPTT	127(15.0)
Sensory threshold determination	70(8.3)
Nerve conduction rate test	35(4.1)
Other	4(0.5)
Peripheral Vascular Disease Screening Methods	
Palpate the dorsal/posterior tibial artery	430(50.9)
Intermittent claudication or rest pain	418(49.5)
Ankle Brachial Index	172(20.4)
Color Doppler ultrasonography	128(15.2)
Angiography	97(11.5)
Other	1(0.1)

### Sample characteristics and association with diabetic foot risk screening behaviors

Table 3 shows the sample characteristics and comparisons of diabetic foot risk screen behaviors among participants with different sample characteristics. Among the 844 general practitioners, 404 were male (47.9%) and 440 (52.1%) were female. The largest proportion concentrated in the 30–39-year-old age group. The average working year was 16.74 ± 9.35 years, with the largest proportion concentrated in the > 20 years group. The largest proportion of people has an education level of an undergraduate degree (57.2%). In terms of diabetic foot risk screening, the largest proportion of people received equal or more than 3 screening training in the last two years (42.5%), had a medium level of screening cognition (62.7%) and thought it strongly necessary to carry out risk screening (61.6%).

As for the major obstacle to diabetic foot risk screening, the most frequently mentioned obstacle was “limited time and energy” (78.6%), followed by “Expenses not covered by health insurance” (57.9%) and “Patient not cooperating” (57.3%). About half of the participants (50.8%) listed “Lack of screening tools” as a major obstacle.

A further comparison of diabetic foot risk screen behavior scores by sample characteristics showed that diabetic foot risk screen behaviors varied significantly by age, work duration, training frequency, cognition, and attitude of risk screening, as well as the four major obstacles of risk screening. Participants with higher training frequency, good level of risk screening cognition, and thought risk screening was strongly necessary had higher scores of diabetic foot risk screening behaviors. Participants who reported “yes” to each of the four major obstacles had significantly lower diabetic foot risk screening scores than those who reported “no”.

**Table 3:** Univariate analysis of diabetic foot risk screening behavior

Item	N (%)	Score	t/F/H	P
Gender			0.163	0.686
Male Female	404(47.9%) 440(52.1%)	61.75 ± 14.60 61.34 ± 14.79		
Age			2.943	0.032
18–29 30–39 40–49 ≥ 50	88(10.4%) 349(41.4%) 304(36.0%) 103(12.2%)	63.86 ± 13.34 60.22 ± 15.63 62.90 ± 14.40 59.99 ± 12.79		
Work duration (years)			2.810	0.025
<5 6–10 11–15 16–20 >20	96(11.4%) 177(21.0%) 156(18.5%) 143(16.9%) 272(32.2%)	65.453 ± 13.12 59.63 ± 14.90 60.45 ± 15.64 61.70 ± 15.33 62.90 ± 13.95		
Education			0.277	0.842
Secondary school and below College Undergraduate Master’s and above	133(15.8%) 222(26.3%) 483(57.2%) 6(0.7%)	60.97 ± 14.74 61.05 ± 13.32 61.89 ± 15.28 63.57 ± 16.45		
Training Frequency (last two years)			34.111	0.000
0 1 2 ≥ 3	218(25.8%) 167(19.8%) 100(11.8%) 359(42.5%)	55.47 ± 14.42 57.64 ± 14.38 63.49 ± 13.47 66.49 ± 13.45		
Screening cognition			32.048	0.000
Poor Medium Good	236(28.1%) 530(62.7%) 78(9.2%)	56.54 ± 14.68 61.31 ± 14.02 65.28 ± 14.82		
Screening attitude			27.262	0.000
No need Necessary Strongly necessary	7(0.8%) 317(37.6%) 520(61.6%)	54.69 ± 14.16 57.24 ± 14.19 64.24 ± 14.37		
Major obstacle				
Expenses not covered by health insurance			5.666	0.018
Major obstacle Expenses not covered by health insurance Yes No	489 (57.9%) 355 (42.1%)	60.51 ± 14.85 62.95 ± 14.37		
Patient not cooperating				
Yes No	484 (57.3%) 360(42.7%)	60.43 ± 14.86 63.02 ± 14.34	6.456	0.011
Limited time and energy				
Yes No	663 (78.6%) 181(21.4%)	60.11 ± 14.52 66.78 ± 14.16	30.371	0.000
Lack of screening tools				
Yes No	429 (50.8%) 415 (49.2%)	58.34 ± 15.56 64.83 ± 12.95	43.207	0.000

### Influencing factors of diabetic foot risk screening behavior

The results showed that training frequency in the last two years, the cognition of diabetic foot risk screening, the attitude toward diabetic foot risk screening, limited time and energy, and lack of screening equipment were significant influencing factors of diabetic foot risk screening behaviors (Table 4). Higher training frequency (*β* = 3.197, *p* < 0.001), higher screening cognition (*β* = 2.947, *p* < 0.001), and more positive screening attitude (*β* = 4.564, *p* < 0.001) were associated with more diabetic foot risk screening behaviors, while limited time and energy (*β*=-5.184, *p* < 0.001) and lack of screening tools (*β*=-6.226, *p* < 0.001) were associated with fewer diabetic foot risk screening behaviors.

**Table 4:** Multiple linear regression analysis of diabetic foot risk screening behavior

Item	Regression coefficients	standard error	standard regression coefficient	t	*P*
	39.267	3.145	-	12.485	0.000
Training Frequency (last two years)	3.197	0.362	0.273	8.840	0.000
Screening cognition	2.947	0.658	0.140	4.481	0.000
Screening attitude	4.564	0.903	0.157	5.055	0.000
Limited time and energy	-5.184	1.106	-0.145	-4.686	0.000
Limited screening tools	-6.226	0.905	-0.212	-6.903	0.000

*R = 0.487, *R*^*2*^=0.237, adjusted *R*^*2*^=0.230, *F* = 12.899, *P* < 0.05;

## Discussion

To our best knowledge, this cross-sectional study is the first to investigate specific diabetic foot risk screening behavior based on the guidelines and its influencing factors among general practitioners. The results showed that the general practitioners’ diabetic foot risk screening behaviors in Changsha, China were at a moderate level, with an average standard score of 61.53 ± 14.69, and only 32.1% of general practitioners reported always or frequently screened their diabetic patients for diabetic feet, which was lower than the reported 69% in South Africa [23]. In fact, 69% of the reported frequency of screening in general practice in South Africa was the result of one year after the implementation of the intervention. Therefore, some targeted interventions are needed to be taken to improve the screening behavior of general practitioners in China.

The ADA recommends components of annual diabetic foot risk screenings should emphasize the importance of peripheral neuropathy and peripheral vascular disease [24]. But our results showed low screening rates in peripheral neuropathy, and peripheral vascular disease, despite high screening rates in other items such as blood sugar, diabetes comorbidities and complications, and general information. It may be due to the screening of diabetic peripheral neuropathy and peripheral vascular disease, which requires the use of certain technology and equipment and requires more time. At the same time, the high screening rates in blood sugar, diabetes comorbidities, medical history, and so on, may be related to the requirement in China’s National Basic Health Service Code for the health management of diabetic patients to routinely ask for medical history, monitor blood glucose, and understand common complications or comorbidities. Therefore, it is necessary to further enhance the awareness of general practitioners on peripheral neuropathy and peripheral vascular disease, and foot skin screening. We recommended that the national health administration should put peripheral neuropathy and peripheral vascular disease into the routine management norms of diabetic patients in primary health care.

Given that peripheral neuropathy and peripheral arterial disease are the two leading causes of diabetic foot, we further investigated the specific methods used by general practitioners to screen for these two lesions. As for the specific methods used in screening peripheral neuropathy, we found generally low rates of 10 g-MF, IPTT, and Tuning fork 128 Hz, although these methods have been demonstrated to be suitable, inexpensive, effective, and easy-to-use tools for foot screening in primary hospitals [25–27]. International Working Group on the Diabetic Foot (IWGDF) [14] “Practical Guidelines on the prevention and management of diabetic foot disease (2019)” also clearly recommends that primary hospitals should choose these screening tools (i.e., 10 g-MF, 128 Hz Tuning fork, and IPTT) to screen for peripheral neuropathy, but less than 25% of general practitioners in this study had used them. Especially for IPTT, previous studies have shown that it is an easy-to-use tool that can effectively screen diabetic foot in the absence of other tools [8], that only 15.0% of general practitioners in this study used IPTT for foot screening. This may be mainly due to the lack of relevant knowledge and training for this research subject. Therefore, the IWGDF guidelines should be further promoted among general practitioners in China, and the training of general practitioners on screening methods should be strengthened to timely update their knowledge and skills.

Good behavior is determined by correct knowledge and a positive attitude [28]. Our study showed that the frequency of training in the last two years, cognition, and attitude toward diabetic foot screening were the main factors influencing screening behaviors. It means that the more training the general practitioners participated in, the higher level of screening cognition they had, and the more positive screening attitudes they held, the better screening behaviors they would have. What is less optimistic is that more than half of the general practitioners had never or rarely received relevant training. Previous studies have also shown that although the medical staff’s willingness to be trained was high, they received insufficient standardized training on diabetic foot from primary medical institutions [29]. This indicates the necessity and importance of increasing general practitioners’ knowledge of diabetic foot screening to improve their screening behaviors. Similar to knowledge-attitude-behavior theory, knowledge is the foundation of behavior, relevant administrative departments, and medical institutions should carry out standardized training related to diabetic foot screening and management, such as establishing standardized simple screening processes and norms, tour lectures on the interpretation of guidelines and consensus and strengthen assessments. This training may improve screening cognition, promote positive screening attitudes, and ultimately improve screening behavior.

In addition, lack of time and energy and limited equipment was also found to be important factors affecting general practitioners’ foot screening. Previous studies in different countries have also shown that limited time during healthcare professionals’ consultations may result in foot assessments being overlooked [30–32], as macrovascular complications and HbA1c monitoring were the primary focus in busy practices. Although the number of general practitioners is constantly growing in China, it still cannot meet the screening needs of large populations of diabetic patients just like in other developing countries, and thus general practitioners may not be able to provide each patient with a full range of services [33]. But how to solve the problem? We suggested, on the one hand, health administrative departments at all levels and community health centers shall strengthen the allocation of general practitioners and include the ratio of manpower in the scope of medical institution evaluation; on the other hand, we should also learn from the successful experience of other countries and train nurse practitioner to undertake diabetic foot screening in combination with national conditions to make up for the shortage of general practitioners [34–36]. In addition, the insufficient screening equipment also reflects the need to further strengthen the quality management of diabetes foot prevention and control in community medical institutions. It is suggested that the provision of basic screening equipment for important complications of diabetes should be included in the terms of inspection and review of community medical institutions. At the meantime, future studies also could develop a more simple and rapid diabetic foot risk screening scale suitable for primary care facilities or develop artificial intelligence technology to help primary care facilities to screen efficiently.

### Strengths and limitation

This study was the first to investigate the specific diabetic foot risk screening behavior base on the guidelines and to analyze its influencing factors among general practitioners. Our finding provides empirical evidence of behavioral responses towards diabetic foot risk screening among general practitioners, the large study sample size and various potential influencing factors examined in the analysis models stand as the strengths of this study.

This study also has several limitations. First, the cross-sectional design makes it impossible to establish causal associations between diabetic foot screening behaviors and their influencing factors. Future longitudinal study designs are needed for causal relationships. Second, the general practitioners in the study were all recruited from Changsha and may not represent general practitioners from other areas of China. A future national multi-center survey is needed to get a more representative sample. It is noteworthy mentioning that the sample size recruited in this study is large and covers all of the community health centers in Changsha, China. Third, the evaluation of general practitioners’ cognition, attitude, and behaviors on performing diabetic foot risk screening was based on participants’ self-report, which may be subject to recall bias and also may not truly reflect their cognition, attitude, and behaviors. Future more objective indicators are needed to get a more accurate assessment. Fourth, we didn’t find any difference in the frequency of training attendance by age of the physician, which may be because we only collected their training information over the last two years instead of since employment to reduce recall bias. The total frequency of training they received since employment may affect their attitudes and behaviors toward diabetic foot risk screening, which warrants further investigation in future research.

## Conclusion

In conclusion, the general practitioners’ diabetic foot risk screening behaviors in Changsha, China were at a moderate level, indicating more efforts are needed to improve screening among general practitioners. Diabetic foot risk screening behaviors were affected by training frequency, the cognition of diabetic foot risk screening, the attitude toward diabetic foot risk screening, limited time and energy, and limited screening equipment. These findings provide important guidance for future intervention programs to improve diabetic foot risk screening behaviors, which may be realized through strengthening training on relevant guidelines and evidence-based screening techniques, improving cognition and attitude, provision of more general practitioners or nurse practitioners, and user-friendly risk screening tools.

## Data Availability

The datasets used and/or analyzed during the current study are available from the corresponding author upon reasonable request.

## Abbreviations

ADA: American Diabetes Association
IWGDF: International Working Group on the Diabetic Foot’s
10g-MF: 10 g monofilament
CVI: Content-related validity
IPTT: Ipswich Touch Test
